# Supported Ni Catalyst for Liquid Phase Hydrogenation of Adiponitrile to 6-Aminocapronitrile and Hexamethyenediamine

**DOI:** 10.3390/molecules23010092

**Published:** 2018-01-04

**Authors:** Chengqiang Wang, Zekun Jia, Bin Zhen, Minghan Han

**Affiliations:** 1 Department of Chemical Engineering, Tsinghua University, Beijing 100084, China; thuwcq@163.com (C.W.); jiazekun1991@163.com (Z.J.); zhenbin@tjut.edu.cn (B.Z.); 2 College of Chemistry and Chemical Engineering, Tianjin University of Technology, Tianjin 300384, China

**Keywords:** supported Ni catalyst, adiponitrile hydrogenation, 6-aminocapronitrile, hexamethyenediamine

## Abstract

Supported Ni catalysts prepared under different conditions, for liquid phase hydrogenation of adiponitrile (ADN) to 6-aminocapronitrile (ACN) and hexamethyenediamine (HMD), were investigated. The highly reactive imine intermediate can form condensation byproducts with primary amine products (ACN and HMD), which decreased the yield coefficient of primary amines. The catalysts support, condition of catalyst preparation and dosage of additive were studied to improve the yield. A highly dispersed Ni/SiO_2_ catalyst prepared by the direct reduction of Ni(NO_3_)_2_/SiO_2_ suppressed the condensation reactions by promoting the hydrogenation of adsorbed imines, and it gave the improved hydrogenation activity of 0.63 mol·kg_cat_^−1^·min^−1^ and primary amine selectivity of 94% when NaOH was added into the reactor.

## 1. Introduction

The hydrogenation of ADN to ACN and HMD is an important industrial process [1,2,3,4], which is mainly catalyzed by Raney Ni catalysts [5,6]. Although the skeleton structure of Raney Ni catalysts favors a high catalytic activity, it causes low mechanical strength and bad regeneration capability. In addition, a safety hazard also exists as the Raney Ni catalysts are flammable when exposed to air. Due to these drawbacks, many researchers have been working on developing supported Ni catalysts to replace Raney Ni. The use of a supported Ni catalyst can dramatically reduce the cost of the catalyst, making the process easy to operate and improving the safety of the hydrogenation reaction. The main challenge is that so far, the supported Ni catalysts are inferior in terms of catalytic activity and primary amine selectivity [7,8].

**Figure 1:**
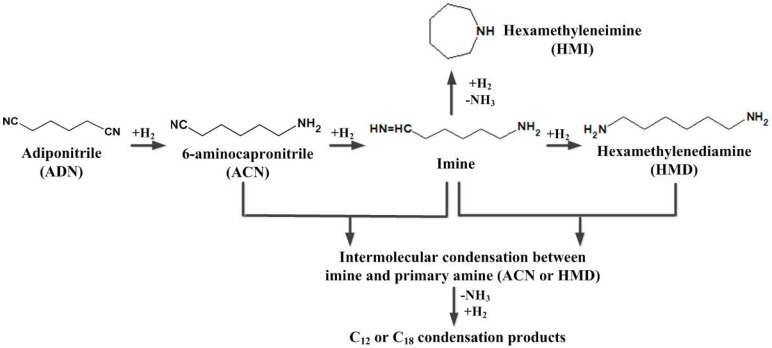
Mechanism of ADN hydrogenation.

The reaction scheme for the hydrogenation of ADN is given in Figure 1 [9,10,11]. ADN is hydrogenated to ACN and then to HMD. During the sequential hydrogenation, a highly reactive imine intermediate is formed, which has a strong tendency towards intermolecular condensation with the primary amine products (ACN and HMD) and intramolecular cyclization to undesired condensation byproducts and hexamethyleneimine (HMI). The formation of the primary amines has to compete with these side reactions. From the chemistry, it can be seen that increasing the hydrogenation activity of the catalyst would be an effective way to suppress the byproducts.

In this work, supported Ni catalysts were studied from several aspects, such as the type of support, conditions of catalyst preparation and dosage of additive, in order to increase the yield of primary amines. A highly dispersed Ni/SiO_2_ catalyst prepared by the direct reduction of a Ni(NO_3_)_2_ precursor [12] was found able to promote the hydrogenation of the imine intermediate so that its performance was close to that of Raney Ni.

## 2. Results and Discussion

### 2.1. Catalyst Support

Since the catalyst support has an important influence on the catalytic performance, the effect of different supports was investigated. Many oxides can be used as the support, among which Al_2_O_3_ and SiO_2_ are the most widely used. The characterization result of the specific surface area and pore structure of the Al_2_O_3_, SiO_2_ and Ni/Al_2_O_3_, Ni/SiO_2_ used are shown in Table 1. The evaluation result in Table 2 showed the Ni/SiO_2_ was better than Ni/Al_2_O_3_ in terms of the selectivity of primary amines and condensation byproducts.

**Figure 2:**
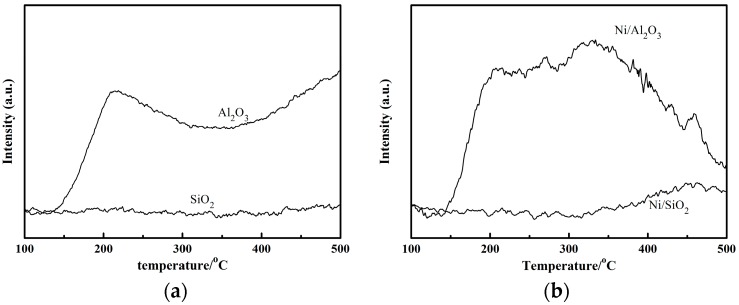
NH_3_-TPD of (**a**) Supports and (**b**) Catalysts.

Using the bifunctional mechanism [13], it is believed that in a nitrile hydrogenation system, the hydrogenation reactions are catalyzed by metal sites while condensation reactions are catalyzed by acid sites. This would indicate that the acidic properties of the support are crucial to the catalytic performance. A stronger acidity favors the conversion of nitrile but decreases the selectivity to primary amines. The NH_3_-TPD data for Al_2_O_3_ and SiO_2_ and their corresponding catalysts are presented in Figure 2a. Al_2_O_3_ has a large NH_3_ desorption peak in the temperature range from 100 to 500 °C while SiO_2_ does not. After the introduction of the Ni, Ni/Al_2_O_3_ still showed an obvious NH_3_ desorption peak in contrast to Ni/SiO_2_ as shown in Figure 2b. The acid sites can absorb imine intermediate and primary amine and cause them to react with each other so that condensation products are produced, which lead to the decreased selectivity of the target products.

Therefore, SiO_2_ is the more suitable support for the hydrogenation of ADN to ACN and HMD, and SiO_2_ was used as the support in the following study.

### 2.2. Direct Reduction Method

The supported Ni catalyst was prepared by the wetness incipient impregnation method, which consists of three steps: impregnation, calcination and reduction. After calcination and reduction, the Ni(NO_3_)_2_ loaded on the supports became metal particles with catalytic activity. The calcination and reduction are key steps and have important influence on the catalytic performance.

**Figure 3:**
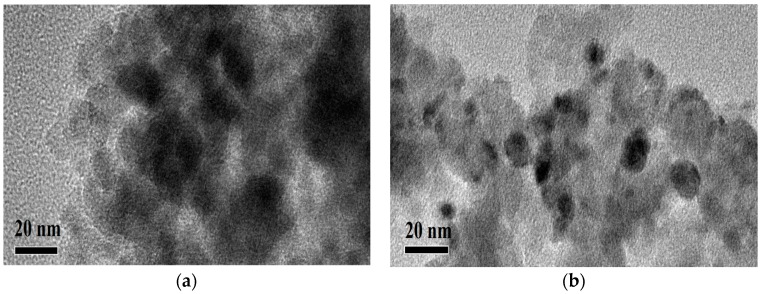
TEM images of (**a**) Ni_CR_/SiO_2_ and (**b**) Ni_DR_/SiO_2_.

Table 3 shows mean particle sizes of Ni estimated by the two independent methods, TEM and XRD analysis [14]. Figure 3 showed the TEM images of two catalysts. The particle size of nickel on the Ni_DR_/SiO_2_ is around 7 nm, while that on the Ni_CR_/SiO_2_ is much larger. XRD analysis results of the catalysts show the similar information, 6.4 and 17.7 nm, respectively.

The H_2_ uptake was 76.2 µmol/g by Ni_DR_/SiO_2_ while that by Ni_CR_/SiO_2_ was 15.4 µmol/g, corresponding to the active nickel areas of 30.0 and 6.0 m^2^/g [15]. We can conclude that Ni_DR_/SiO_2_ had a superior Ni dispersion of 4.50% while that of Ni_CR_/SiO_2_ is 0.90%, which is in accordance with the TEM and the XRD analysis. Ni(NO_3_)_2_/SiO_2_ catalyst precursor would be totally reduced at 700 °C from H_2_-TPR profiles. The H_2_-chemisorption data showed the H_2_ adsorption capacity of a catalyst partially reduced at 450 °C. This means that H_2_-chemisorption data here cannot be used to calculate the mean particle size accurately.

**Figure 4:**
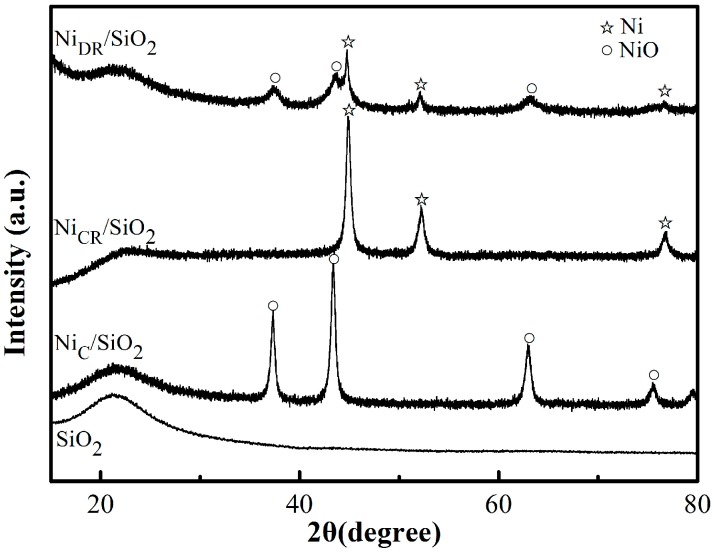
XRD pattern of SiO_2_, Ni_C_/SiO_2_, Ni_CR_/SiO_2_ and Ni_DR_/SiO_2_.

Figure 4 showed the XRD patterns of the catalysts. The characteristic diffraction peaks of NiO in Ni_C_/SiO_2_ are obvious, which indicated that the nickel mainly existed in the form of nickel oxide. After the reduction in H_2_, Ni_CR_/SiO_2_ showed a very sharp Ni peak while Ni_DR_/SiO_2_ showed both NiO and Ni diffraction peaks. Louis et al. [16] have studied supported Ni catalyst and found existence of nickel phyllosilicates, which were located at the surface of catalyst precursor after nickel nitrate impregnated onto silica. The nickel phyllosilicates act as anchoring sites for metallic Ni particles and suppress the formation of large Ni particles. However, calcination before reduction leads to the formation of larger metal particles and to their aggregation. This is probably due to the partial decomposition of nickel phyllosilicates during calcination, the breaking of the anchoring sites for impregnated nickel, which favors nickel migration. Direct reduction of Ni(NO_3_)_2_/SiO_2_ was a moderate reduction process, which sacrificed the reduction degree but gave a superior Ni dispersion.

As described above, the two catalysts prepared by the different methods had different microstructures, which probably caused their catalytic performance for hydrogenation of ADN to be different. Table 4 shows the evaluation result of the catalysts. Ni_DR_/SiO_2_ exhibited a better hydrogenation activity of 0.56 mol·kg_cat_^−1^·min^−1^ compared to Ni_CR_/SiO_2_ with 0.25 mol·kg_cat_^−1^·min^−1^. The selectivity to the primary amine products over Ni_DR_/SiO_2_ was higher than that over Ni_CR_/SiO_2_, which, respectively, were 79% and 54%. Meanwhile, the selectivity to condensation byproducts were inhibited over Ni_DR_/SiO_2_, which decreased from 37% to 3%. In the sequential reaction, shown in Figure 1, the highly reactive imine intermediate can further react along three pathways: further hydrogenation, intramolecular condensation to HMI, and intermolecular to condensation byproducts. The three pathways are parallel, that is, the formation of primary amine competes with the side reactions. Ni_DR_/SiO_2_ exhibited a superior hydrogenation activity, which promoted the hydrogenation and improved the selectivity to ACN and HMD.

**Figure 5:**
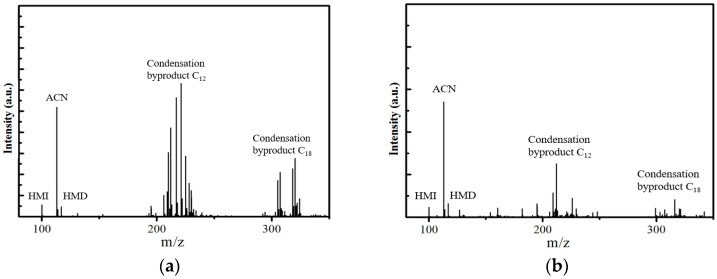
Mass spectra of (**a**) Ni_CR_/SiO_2_ and (**b**) Ni_DR_/SiO_2_.

The condensation byproducts were secondary and tertiary amines with a high molecular weight which were not eluted and cannot be detected in the GC analysis. The product mixture was analyzed by a mass spectrometer to confirm the existence of condensation byproducts. The result is shown in Figure 5. The mass to charge ratio of protonated HMI, ACN and HMD are, respectively, *m*/*z* = 100, 113, 117. The peaks of *m*/*z* = 200~300 indicated the formation of C_12_ and C_18_ oligomers. These showed clearly that the condensation byproducts from Ni_DR_/SiO_2_ was decreased, which verified the GC evaluation result.

Ni_DR_/SiO_2_ had a higher H_2_ adsorption capacity from H_2_ uptake result, which would indicate that amines and imine intermediates are more abundantly adsorbed on Ni_DR_/SiO_2_. The C atoms of imines were subjected to nucleophilic attack by N atoms of the amines [17], leading to the formation of a secondary imine which can be further hydrogenated to HMI. Ni_DR_/SiO_2_ was more selective for intramolecular side reaction. As seen in Table 4, the selectivity to HMI over Ni_CR_/SiO_2_ was 9% while that over Ni_DR_/SiO_2_ was 18%. From this point, further developing of the supported Ni catalyst prepared by the direct reduction method is still necessary. The conditions of catalyst preparation and reaction conditions have to be optimized to inhibit the formation of HMI and improve the selectivity to primary amines.

### 2.3. Reduction Teamperature

**Figure 6:**
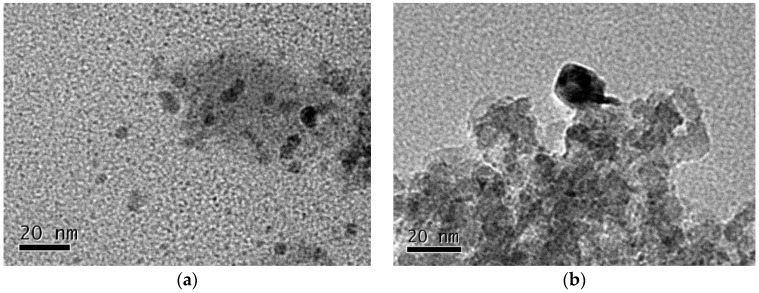

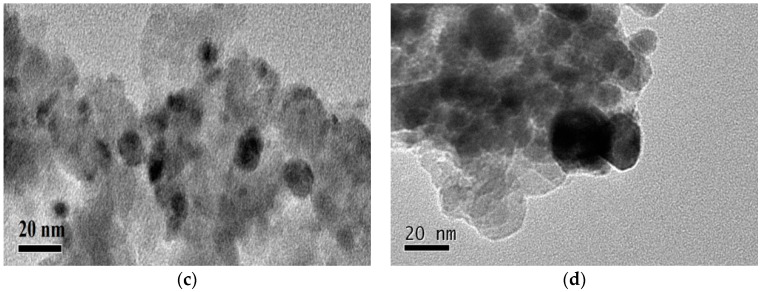
TEM images of the catalysts reduced at different temperatures: (**a**) 350 °C; (**b**) 400 °C; (**c**) 450 °C; (**d**) 500 °C.

The microstructure of supported Ni catalyst prepared by direct reduction method is affected by reduction temperature significantly. The TEM images of catalysts reduced at different temperature are shown in Figure 6. The mean particle sizes of Ni became larger with the increase of reduction temperature obviously.

**Figure 7:**
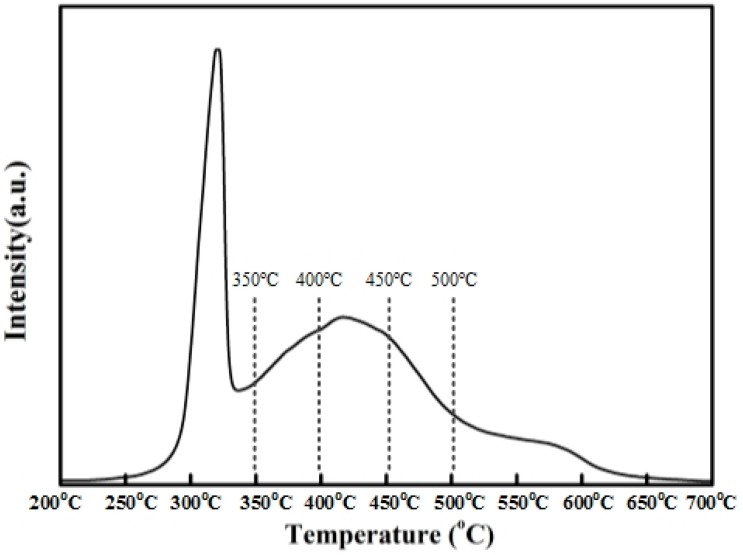
H_2_-TPR of catalyst precursors.

The H_2_-TPR profiles of Ni(NO_3_)_2_/SiO_2_ were given in Figure 7. The TPR profiles of the impregnated catalysts, performed without any previous calcination, exhibit three peaks of hydrogen consumption at 300, 400, and about 550 °C, attributed to the decomposition of nickel nitrate into NiO, the reduction of NiO and the reduction of nickel phyllosilicates. After TPR up to 700 °C, the nickel is totally reduced. Before complete reduction, the nickel phyllosilicates are probably located at the interface between silica and the remaining nickel, for which they act as anchoring sites. During TPR, after nitrate decomposition, the NiO particles located on the phyllosilicates are reduced into Ni^0^ at 400 °C without any migration. Between 400 and 700 °C, the increase in metal particle size is due not only to NiO migration induced by thermal effect but also to the reduction of the phyllosilicates by the weakening of the anchoring strength.

The H_2_-TPR profiles of Ni(NO_3_)_2_/SiO_2_ are in accordance with the TEM analysis of the four catalysts reduced at 350, 400, 450 and 500 °C, respectively.

**Figure 8:**
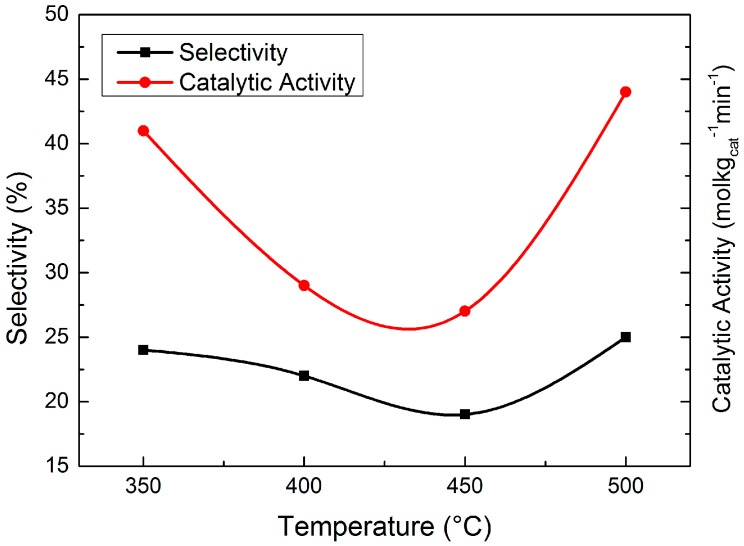
Evaluation result of catalysts reduced at different temperatures.

Figure 8 presents the evaluation result of the four catalysts. The catalytic activity first decreased and then increased with the increase of reduction temperature, which can be explained by the reduction degree and Ni dispersion. With the increase of reduction temperature, the reduction degree of the catalysts became higher while the Ni dispersion became lower because the nickel phyllosilicates were gradually decomposed. Ni/SiO_2_ reduced at 350 °C had a high hydrogenation activity because of its high Ni dispersion. When the reduction temperature was increased to 400 °C and 450 °C, the migration and aggregation of Ni particles led to a lower metallic Ni dispersion, which caused the catalytic activity decrease. Although the Ni dispersion of Ni/SiO_2_ reduced at 500 °C became even lower, the catalytic activity became higher because of its higher reduction degree. In conclusion, Ni dispersion was the key factor to affect the catalytic activity for the reduction temperature range from 350 °C to 450 °C while the reduction degree was the key factor in the temperature range from 450 °C to 500 °C, which explained the variation of catalytic activity.

450 °C was considered the optimum temperature for the reduction when both catalytic activity and selectivity to HMI were taken into account.

### 2.4. Additive

Most of the side reactions are deamination reactions as shown in Figure 1. Thermodynamically, adding an alkaline substance as additive to the liquid reaction system would benefit the catalytic activity and selectivity to primary amines [5,18]. In ADN hydrogenation, H_2_ and nitrile adsorbed on metallic Ni first and then they reacted with each other to imine intermediates and amines. Imines and amines desorbed from the catalyst with difficulty [19], which would not only suppress the following hydrogenation of nitriles, but also promote the condensation side reactions. The addition of an alkaline substance increases the electron density of metallic Ni on the catalyst surface, which would benefit the desorption of imine and amine [20].

**Figure 9:**
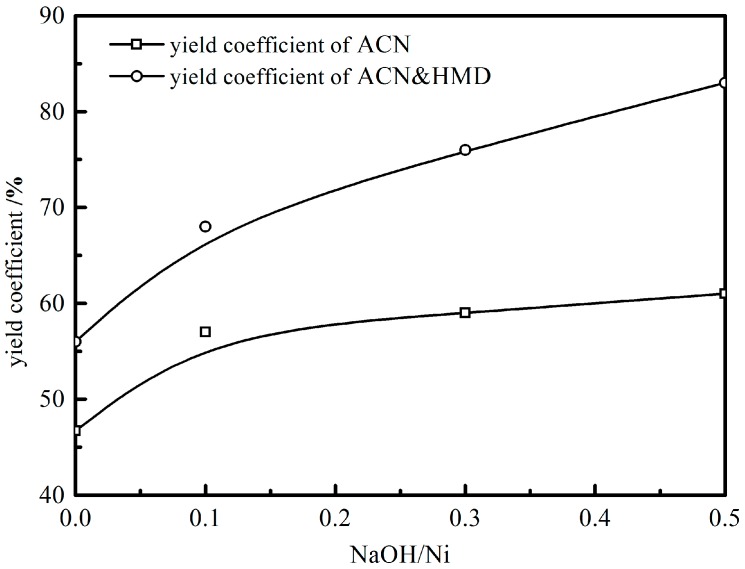
Evaluation result of catalysts with the use of different NaOH/Ni ratios.

NaOH was chosen as an additive to improve the Ni_DR_/SiO_2_ catalyst. The evaluation result is presented in Figure 9 and Table 5. The mass ratio NaOH/Ni was used as the independent variable. With NaOH/Ni increasing, the catalytic activity rose from 0.39 mol·kg_cat_^−1^·min^−1^ to 0.63 mol·kg_cat_^−1^·min^−1^ and the selectivity to condensation byproducts decreased from 12% to 1% while that to HMI decreased from 21% to 5%. When the NaOH/Ni was raised to 0.5, the primary selectivity was as high as 94%. Clearly, the effect of NaOH on the catalytic activity and product selectivity was positive.

The addition of NaOH improved the hydrogenation activity and improved the target products selectivity significantly. After the introduction of NaOH, the performance of Ni_DR_/SiO_2_, reduced at 450 °C, is as excellent as Raney Ni, which is presented in Table 6.

## 3. Materials and Methods 

### 3.1. Catalyst Preparation

The supported Ni catalysts were prepared by incipient wetness impregnation, which included three steps: impregnation, calcination and reduction. Ni/SiO_2_ catalyst prepared by the direct reduction (DR) of Ni(NO_3_)_2_/SiO_2_ in H_2_ was denoted as Ni_DR_/SiO_2_. For comparison, another catalyst prepared by calcination and reduction (CR) was denoted as Ni_CR_/SiO_2_. They were prepared as follows. The loading amount of nickel was 20 wt.%.

Ni_DR_/SiO_2_: First, SiO_2_ (Alfa Aesar, Haverhill, MA, USA) was soaked in an aqueous solution of Ni(NO_3_)_2_ (98%, Alfa Aesar) at room temperature. Then, the mixture was treated with ultrasonic waves for 2 h and dried at 80 °C for 12 h. Finally, the catalyst precursor was reduced in H_2_ flow at 450 °C for 8 h.

Ni_CR_/SiO_2_: The impregnated and dried Ni(NO_3_)_2_/SiO_2_ was calcined at 450 °C for 4 h and then reduced in H_2_ flow. The other steps were the same as those for Ni_DR_/SiO_2_. Before reduction, the calcined (C) catalyst was denoted as Ni_C_/SiO_2_.

### 3.2. Catalyst Characterization

The specific surface area and pore structure of support and catalyst were determined by N_2_ adsorption with a Quadrasorb SI instrument. The crystalline phase of the catalysts was characterized by a Bruker Advance D8 X-ray diffractometer with a Cu Kα radiation source. The H_2_ uptake of the catalysts was determined by H_2_ chemisorption on a Quantachrome ChemBET Pulsar TPR/TPD instrument. The active Ni area was calculated assuming H/Ni_surface_ = 1 and a surface area of 6.5 × 10^−20^ m^2^ per Ni atom. A JEM-2010 transmission electron microscope was employed to examine the Ni particle size of catalysts. Some product samples were analyzed by mass spectrometry (MS model: Q Exactive) to identify the relative amount of condensation byproducts.

### 3.3. Catalytic Reaction

The hydrogenation of ADN was carried out in a stainless-steel autoclave equipped with a temperature control system and magnetic stirrer. Typically, 5 g ADN (98%, Alfa Aesar, Haverhill, MA, USA), 80 mL of methanol (>99.5%, Beijing Chemical Works, Beijing, China), 5 g pre-reduced catalyst and 0.1 g NaOH (>96.0%, Beijing Chemical Works) were added into the reactor. The hydrogenation conditions were: 80 °C, 3 MPa, 500 rpm. The products were sampled online and analyzed by a gas chromatograph (GC 7890F, Techcomp Instrument Company, Kwai Chung, Hong Kong) equipped with a flame ionization detector and a KB-624 capillary column (30 m × 0.32 mm × 1.8 μm, Kromat). The condensation byproducts were high molecular weight secondary and tertiary amines which were not eluted in the GC. Phenylamine was used as the internal standard to determine the content of ADN, ACN, HMD and HMI. The catalytic activity, the conversion of ADN and the selectivity to ACN and HMD were calculated as:

(1) $$\mathrm{Catalytic}\;\mathrm{Activity}\;=\;\frac{\mathrm{moles}\;\mathrm{of}\;\mathrm{converted}\;\mathrm{ADN}}{{\mathrm{Time}\;\times \;m}_{\mathrm{Cat}}}$$

(2) $$\mathrm{ADN}\;\mathrm{Conversion}\;=\;\frac{\mathrm{moles}\;\mathrm{of}\;\mathrm{converted}\;\mathrm{ADN}}{\mathrm{moles}\;\mathrm{of}\;\mathrm{ADN}\;\mathrm{feedstock}}\;\times \;100\%\;$$

(3) $$\mathrm{ACN}\;\mathrm{Selectivity}\;=\;\frac{\mathrm{moles}\;\mathrm{of}\;\mathrm{ACN}}{\mathrm{moles}\;\mathrm{of}\;\mathrm{converted}\;\mathrm{ADN}}\;\times \;100\%$$

(4) $$\mathrm{HMD}\;\mathrm{Selectivity}\;=\;\frac{\mathrm{moles}\;\mathrm{of}\;\mathrm{HMD}}{\mathrm{moles}\;\mathrm{of}\;\mathrm{converted}\;\mathrm{ADN}}\;\times \;100\%$$

## 4. Conclusions

Supported Ni catalysts for the liquid phase hydrogenation of ADN were investigated. The following conclusions are based on the results and discussion:

1. The support affects the catalytic performance of supported Ni catalysts by providing original acid sites, which promote the condensation side reactions and decrease the selectivity to primary amines. SiO_2_ is chosen as the support in this work providing less original acid sites.
2. The catalyst prepared by the direct reaction method, without a prior calcination step, gave smaller Ni particles, this is, a higher Ni dispersion and H_2_ uptake capacity, which improved the catalytic activity and inhibited the intermolecular condensation side reaction.
3. The reduction temperature had an appreciable effect on the catalytic performance of Ni_DR_/SiO_2_. 450 °C was regarded as a preferable temperature to suppress the intramolecular condensation reaction to HMI.
4. The addition of NaOH into the reaction system improved the hydrogenation activity and significantly improved the target products selectivity. When the NaOH/Ni mass ratio was increased to 0.5, the selectivity to primary amines attained 94%.

## Figures and Tables

**Table 1:** Specific surface area and pore structure of the supports and the catalysts.

Sample	Specific Surface Area/m^2^g^−1^	Pore Volume/cm^3^·g^−1^	Average Pore Size/nm
Al_2_O_3_	242	0.64	9.9
Ni/Al_2_O_3_	175	0.41	9.5
SiO_2_	245	0.94	17.8
Ni/SiO_2_	190	0.72	17.4

**Table 2:** Evaluation reaction result of Ni/SiO_2_ and Ni/Al_2_O_3_.

Catalysts	Catalytic Activity/mol·kg_cat_^−1^·min^−1^	Selectivity/%			
		ACN	HMD	HMI	Condensation Byproducts
Ni/SiO_2_	0.25	50	4	9	37
Ni/Al_2_O_3_	0.29	43	4	8	45

**Table 3:** Ni particle size of Ni_CR_/SiO_2_ and Ni_DR_/SiO_2_ from TEM and XRD analysis.

Sample	Ni Particle Size(a) ^1^/nm	Ni Particle Size(b) ^2^/nm
Ni_CR_/SiO_2_	19	17.7
Ni_DR_/SiO_2_	7	6.4

^1^ Estimated by TEM analysis. ^2^ Calculated by XRD analysis according to Scherrer equation.

**Table 4:** Evaluation reaction result of Ni_CR_/SiO_2_ and Ni_DR_/SiO_2_.

Catalysts	Catalytic Activity/Mol·kg_cat_^−1^·min^−1^	Selectivity/%			
		ACN	HMD	HMI	Condensation Byproducts
Ni_CR_/SiO_2_	0.25	50	4	9	37
Ni_DR_/SiO_2_	0.50	66	13	18	3

**Table 5:** Evaluation reaction result of the catalysts with different NaOH/Ni mass ratio.

NaOH/Ni	Catalytic Activity/mol·kg_cat_^−1^·min^−1^	Selectivity/%			
		ACN	HMD	HMI	Condensation Byproducts
0	0.39	55	12	21	12
0.1	0.50	66	13	18	3
0.3	0.56	70	21	7	2
0.5	0.63	70	24	5	1

**Table 6:** Evaluation reaction result of the Ni_DR_/SiO_2_ and Raney Ni.

Catalysts	Catalytic Activity/mol·kg_cat_^−1^·min^−1^	Selectivity/%			
		ACN	HMD	HMI	Condensation Byproducts
Raney Ni	0.61	78	17	1	4
Ni_DR_/SiO_2_	0.63	70	24	5	1
